# Giant cell tumor of bone with H3F3B mutation: A case report

**DOI:** 10.1097/MD.0000000000032995

**Published:** 2023-02-17

**Authors:** Ruinuan Wu, Xikang Wu, Xin Weng, Yingjie Xiu, Gang Xu, Xiajing Liu, Xia Liu

**Affiliations:** a Department of Pathology, Shenzhen Second People’s Hospital, Shenzhen, China; b Department of Diagnostic Bone Oncology, Shenzhen Second People’s Hospital, Shenzhen, China; c Department of Diagnostic Imaging, Shenzhen Second People’s Hospital, Shenzhen, China.

**Keywords:** Giant cell tumor, *H3F3A*, *H3F3B*

## Abstract

Giant cell tumor of bone is a locally aggressive and rarely metastasizing neoplasm that typically affects the ends of long bones or the axial skeleton of young to middle-aged adults. As many as 69% to 100% of giant cell tumors harbor *H3F3A* gene mutations, while *H3F3B* gene mutations have rarely been reported.

**Patient concerns:**

A 53-year-old male patient who underwent right distal femoral tumor resection.

**Diagnoses:**

Preoperative CT plain scan indicated giant cell tumor of bone with pathological fracture. Laboratory findings were as follows: serum calcium was 2.23 mmol/L (reference range: 2.1–2.55 mmol/L) and serum phosphorus was 1.35 mmol/L (reference range: 0.81–1.45 mmol/L).

**Interventions:**

The histological morphology showed the typical features of a conventional GCT. The immunoprecipitation analysis results were as follows: H3.3G34W(−), H3.3G34R(−), H3.3G34V(−), and H3K36M(−). Sanger sequencing showed that the *H3F3A* and *H3F3B* gene mutations were wild type. The high-throughput gene sequencing results revealed the *H3F3B* gene mutations H3.3p.Gly35Trp and H3.3p.Val36Leu.

**Outcomes:**

The patient was stable with no recurrence in 12 months follow-up.

**Lessons:**

Giant cell tumor of bone with *H3F3B* gene mutations is extremely rare. In the pathological diagnosis of bone tumors, we need to analyze clinical presentation, imaging features, histology, immunophenotype, and cytogenetic/molecular alterations, in order to get a correct diagnosis.

## 1. Introduction

Giant cell tumors of bone represent 4% to 5% of primary bone tumors. The peak age of onset is 20 to 45 years, and a slight female predominance was reported in China.^[1–3]^ It typically affects the ends of long bones and often involves the metaphysis.^[4]^ Radiographically, giant cell tumors of bone often show expansile, eccentric, lytic lesions with well-defined margins, and nonsclerotic borders and no matrix mineralization.^[5]^ Histologically, giant cell tumors are typically composed of neoplastic mononuclear stromal cells and osteoclast-like giant cells. Mononuclear cells appear to grow in syncytium, have ill-defined cell borders and have little eosinophilic cytoplasm. Mononuclear cell nuclei are round to ovoid, vesicular, have 1 to 2 central nucleoli, and atypical mitotic figures are absent. The giant cells have a variable number of nuclei, some with > 50 per cell, and are morphologically identical to nuclei of mononuclear cells. Other secondary changes commonly encountered include necrosis, hemorrhage, haemosiderin deposition, aneurysmal change, collections of foamy macrophages, reactive/metaplastic bone formation around the tumor. Immunohistochemically, as many as 90% of cases express H3.3G34W, and the expression of H3.3G34V and H3.3G34R has also been reported.^[6]^ An H3F3A gene mutation is detected in neoplastic stromal cells in as many as 69% to 100% of giant cell tumors.^[7,8]^ The mechanism by which mutant H3.3 drives the neoplasms is under study. Pavlo Lutsik found that H3.3G34W is incorporated into the chromatin and associates with massive epigenetic alterations on the DNA methylation, chromatin accessibility and histone modification level. These lead to globally altered epigenome regulation and, thereby, to an impairment of differentiation.^[9]^

## 2. Case presentation

**Figure 1.**
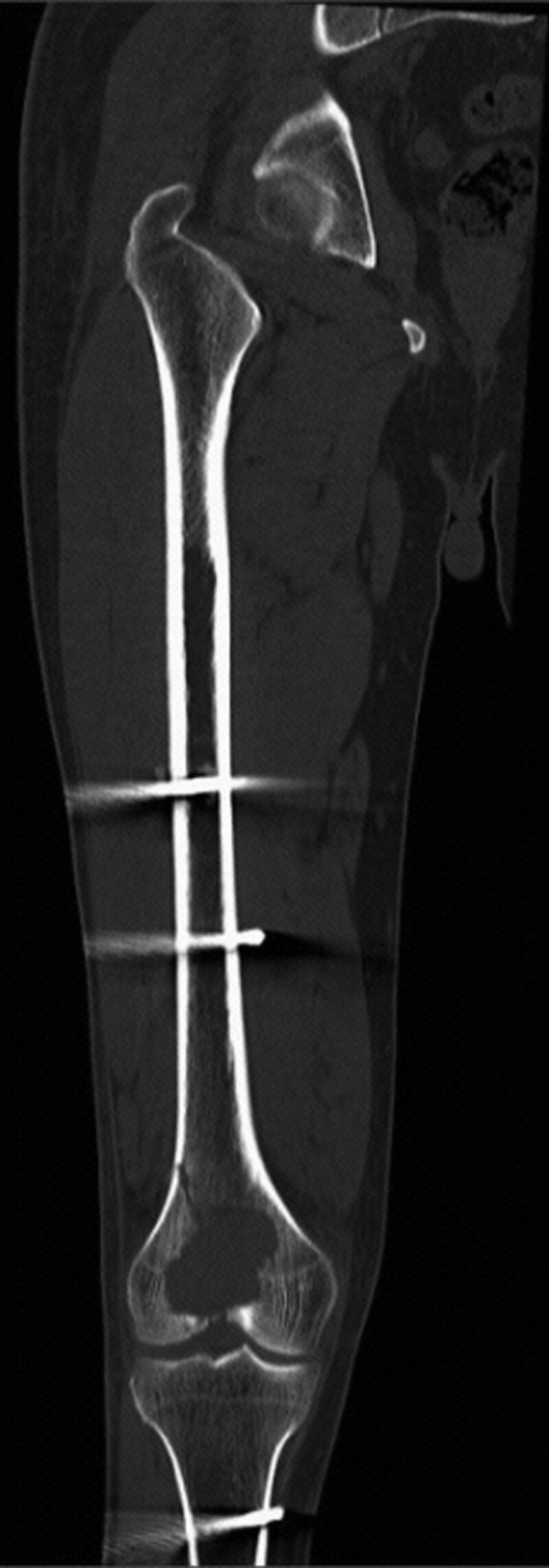
Plain scan CT showed an expansile and well-defined bone destruction in the right distal femur, without calcification, ossification shadow or sclerotic border. Multiple fracture lines were seen around.

**Figure 2.**
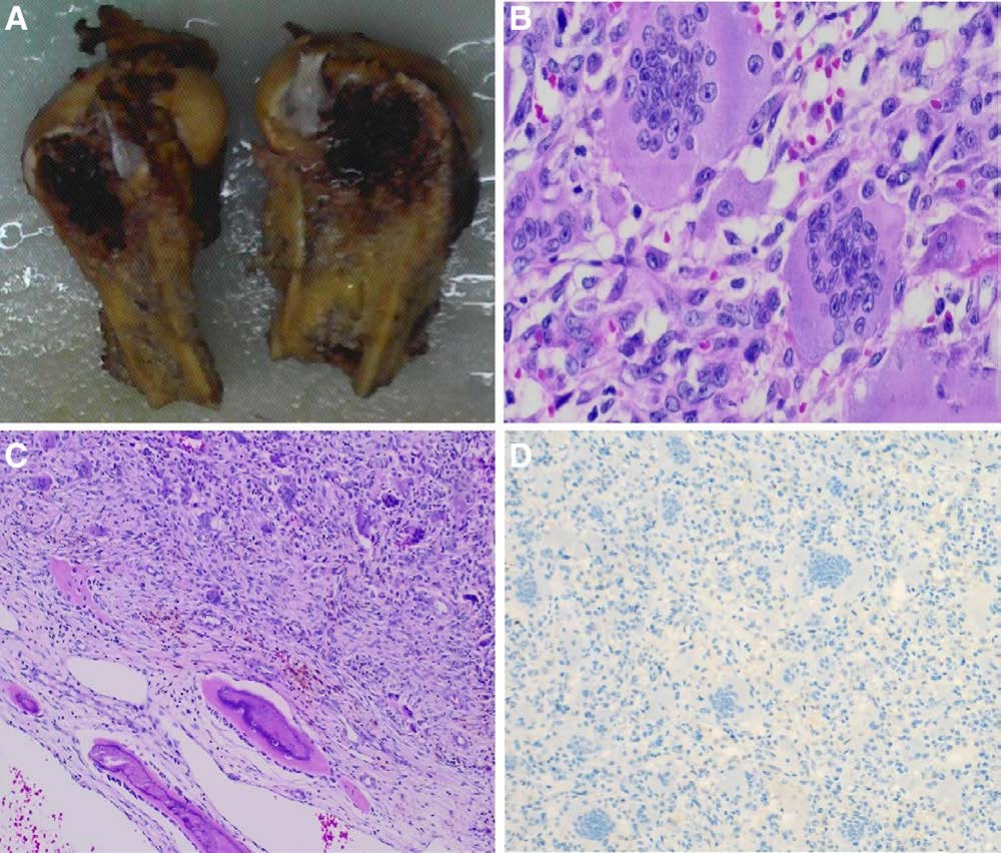
Postoperative pathological and immunohistochemical staining results. 2A Mass specimen. 2B Microscopically, the mass was composed of mononuclear stromal cells and osteoclast-like giant cells. HE 400×. 2Creactive/metaplastic bone formation were seen around the lesion. HE 100×. 2DTumor cells were negative for H3.3G34W. IHC 100×.

**Figure 3.**
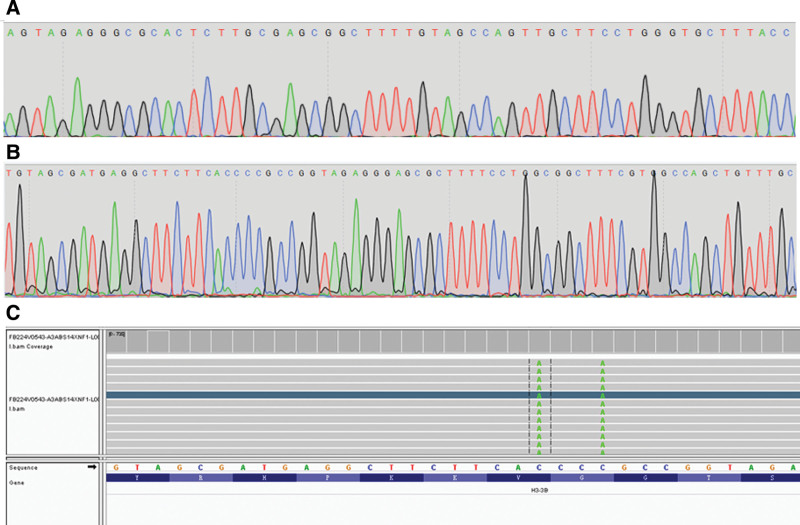
A Sanger sequencing showed that the *H3F3A* gene was wild type. 3B Sanger sequencing showed that the *H3F3B* gene was wild type. 3C Genehigh-throughput sequencing results revealed *H3F3B* gene mutations, including H3.3p.Gly35Trp and H3.3p.Val36Leu.

A 53-year-old male patient sustained a right femur fracture by falling down stairs and underwent femur fracture fixation at an outside hospital. Giant cell tumor of bone was considered based on the postoperative biopsy pathological analysis. The patient was transferred to our hospital on April 9, 2022, for continued treatment. Plain scan CT in our hospital showed expansile bone destruction in the right distal femur, in which a thin bone crest could be seen, without calcification or ossification shadow (Fig. 1). The lesion showed a well-defined, nonsclerotic border with multiple fracture lines. Imaging analysis indicated giant cell tumor of bone with pathological fracture. Laboratory findings were as follows: serum calcium was 2.23 mmol/L (reference range: 2.1–2.55 mmol/L) and serum phosphorus was 1.35 mmol/L (reference range: 0.81–1.45 mmol/L). The biopsy section from the outside hospital showed that the lesion was composed of mononuclear stromal cells and osteoclast-like giant cells. The mononuclear cells had an ill-defined cell membrane with pale eosinophilic cytoplasm. The nuclei were round or ovoid, with open chromatin with one or two small nucleoli, and mitotic count was not seen. The multinucleated giant cells were numerous and evenly distributed among the mononuclear cells. The number of nuclei ranged from 10 to 40, and their morphology resembled that of mononuclear nuclei. Immunohistochemical staining was negative for the H3.3G34W marker in our hospital. Sanger sequencing showed that the H3F3A gene mutation was wild type. The pathology consultation diagnosis was giant cell tumor of bone. This diagnosis was made in conjoint assessment by radiologists, pathologists, and orthopedic surgeons in a multidisciplinary team meeting before surgery. The lesion of the right femoral tumor segment was resected. The size of the articular bone tissue was 11 cm × 8 cm × 7 cm, with a dark reddish soft area at the bone ends measuring approximately 7 cm × 4 cm × 3.5 cm (Fig. 2A). The morphology of the surgical excision was similar to that of the biopsy specimen (Fig. 2B). Recent hemorrhage, haemosiderin deposition, fibroplasia were observed in the tumor stroma, and reactive/metaplastic bone formation was observed around the lesion (Fig. 2C). Immunohistochemical markers of mononuclear stromal cells were positive for SATB2 but negative for H3.3G34W(Fig. 2D), H3.3G34R, H3.3G34V, and H3K36M. Sanger sequencing showed that the H3F3A and H3F3B gene mutations were wild type (Fig. 3A and B). No USP6 rearrangement was detected by FISH analysis. Gene high-throughput sequencing results revealed the missense mutation c.103G > T (Gly35Trp) in exon 2 and the missense mutation c.106G > T (Val36Leu) in exon 2 of the H3F3B gene (Fig. 3C). Combined with clinical, radiographic, and histopathologic morphology, we still retain our original diagnosis of giant cell tumor of the bone after obtaining the sequencing results. The patient was stable with no recurrence at the date of submission.

## 3. Discussion

H3.3 p.Gly34Trp (G34W) is now identical to H3.3 p.Gly35Trp (G35W) at the sequencing level, so the H3F3B/H3.3 p.Gly35Trp (G35W) and H3.3 p.Val36Leu (V36L) mutations are identical to H3.3 p.G34W and H3.3 p.V35L. Both H3F3A and H3F3B encode identical conserved H3.3 histone proteins that differ only in their mRNA untranslated regions and regulatory sequences.^[10]^ Therefore, in our case, the H3F3B “G34W” mutation is analogous to the H3F3A “G34W” mutation in conventional giant cell tumors of bone. The vast majority of giant cell tumors of bone contain H3F3A gene mutations. However, Arjen H.G. Cleven reported 2 different variants of H3F3B mutations, including S28N and G34L, instead of H3F3A. A similar phenomenon is reported in chondroblastomas, in which the majority of cases have the H3F3B “K36M” mutation and a minor subset have the H3F3A “K36M” mutation. Sam Behjati and his colleague suspected that chondroblastomas and giant cell tumors of bone are derived from a common precursor cell.^[11]^ This may explain why these tumors have intersecting genetic mutations. Moreover, Leilei Shi and her colleague have revealed that histone H3.3 G34 mutations alter histone K36 methylation in cis.^[12]^ V35 is in close proximity to G34, it is conceivable that G34 mutations may impact V35. This hypothesis requires further study. The detection of H3F3B was wild type by Sanger sequencing but mutant type by high-throughput sequencing. Thus, we conjecture that because of the heterogeneous cell population in tumor cells, only a small proportion of the isolated DNA will originate from the neoplastic stromal cells that harbor the mutation. In addition, we worked with decalcified tissue, which renders lower DNA quality, as shown by Cleven.^[7]^ The sensitivity is increased by using targeted next-generation sequencing, as shown by Presneau et al.^[13]^ Furthermore, the H3F3B gene mutations were c.103G > T (Gly35Trp) in exon 2, with an abundance of only 15.0%, and c.106G > T (Val36Leu) in exon 2, with an abundance of only 15.4%. Therefore, false negatives cannot be excluded by Sanger sequencing.

Of course, osteoclastic giant cell-rich tumors need to be differentiated from other diagnoses. Chondroblastoma should be considered because the patient harbored a H3F3B gene mutation, and > 95% of chondroblastoma patients harbor H3F3B gene mutations.^[11]^ However, chondroblastomas usually involve the epiphyseal region of long bones in patients with an immature skeleton; however, in adult patients aged > 30 years, short tubular bones and flat bones, rather than long bones, are more commonly affected.^[14,15]^ Radiographically, spotty calcifications are often observed, and sclerotically marginated lucent lesions are usually present in patients with chondroblastoma.^[4]^ Histologically, chondroblastoma is composed of ovoid to polygonal mononuclear cells with well-defined cell membranes and interspersed osteoclast-like giant cells. The nuclei of mononuclear cells are reniform or coffee bean-shaped, the cytoplasm is eosinophilic. The number of giant cells is less, they are less evenly distributed than those in giant cell tumors. Additionally, they are often numerous in areas of matrix production and hemorrhage. Therefore, this case does not support the diagnosis of chondroblastoma based on the clinical, radiographical, and histological features. Moreover, p.Lys36Met substitutions in chondroblastoma are more frequent in the H3F3B gene on chromosome 17,^[11,13]^ while in this case, the H3F3B gene mutations were c.103G > T (Gly35Trp) in exon 2 and c.106G > T (Val36Leu) in exon 2. The solid subtype of aneurysmal bone cyst usually arises in the metaphysis of long bones. On MRI, characteristic fluid–fluid levels are present. Tumours are composed of fibroblast-like spindle cells, osteoclastic giant cells, and reactive bone formation. USP6 rearrangements are present in approximately 70% of aneurysmal bone cysts.^[16]^ For brown tumors, multiple tumors are present, and they commonly affect the pelvis, ribs, clavicles, and extremities. Laboratory tests revealed elevated parathyroid hormone levels, hypercalcaemia, and hypophosphatemia. Radiographic findings may be similar to giant cell tumors of bone, and osteoporosis is evident. Tumours generally have a multinodular architecture formed by fibrous septa dividing the mass into adjacent lobules. The lobules are composed of spindle fibroblasts, extravasated red blood cells and numerous scattered giant cells that cluster in areas of hemorrhage.

Giant cell tumor of bone with *H3F3B* gene mutations is extremely rare. Whether it has its own unique clinicopathological features and prognosis still needs follow-up and the examination of additional data. Combined with the clinical features, the radiographic features and pathologic morphology are the basis for diagnosing primary tumors of the bone. With the development of molecular genetics, many cases with atypical clinical, radiographical, and pathological features need to undergo immunohistochemical and genetic testing. We believe that the careful integration of clinical presentation, imaging features, histology, immunophenotype, and cytogenetic/molecular alterations is crucial for the accurate diagnosis of bone tumours.

## Author contributions

**Data curation:** Xikang Wu, Xin Weng, Yingjie Xiu.

**Formal analysis:** Xikang Wu.

**Resources:** Ruinuan Wu, Gang Xu, Xiajing Liu.

**Writing – original draft:** Ruinuan Wu.

**Writing – review & editing:** Xia Liu
